# Insights and genetic features of extended-spectrum beta-lactamase producing *Escherichia coli* isolates from two hospitals in Ghana

**DOI:** 10.1038/s41598-022-05869-6

**Published:** 2022-02-03

**Authors:** Samiratu Mahazu, Wakana Sato, Alafate Ayibieke, Isaac Prah, Takaya Hayashi, Toshihiko Suzuki, Shiroh Iwanaga, Anthony Ablordey, Ryoichi Saito

**Affiliations:** 1 Department of Molecular Microbiology, Tokyo Medical and Dental University, Tokyo, Japan; 2 Department of Environmental Parasitology, Tokyo Medical and Dental University, Tokyo, Japan; 3 Department of Molecular Virology, Tokyo Medical and Dental University, Tokyo, Japan; 4 Department of Bacterial Pathogenesis, Tokyo Medical and Dental University, Tokyo, Japan; 5 Department of Bacteriology, Noguchi Memorial Institute for Medical Research, University of Ghana, Accra, Ghana

**Keywords:** Bacteriology, Microbial genetics, Antimicrobial resistance

## Abstract

Recently, the emergence and rapid dissemination of extended-spectrum beta-lactamase (ESBL)-producing bacteria, particularly of the family Enterobacteriaceae, has posed serious healthcare challenges. Here, we determined the antimicrobial susceptibility and genetic characteristics of 164 *Escherichia coli* strains isolated from infected patients in two hospitals in Ghana. In total, 102 cefotaxime-resistant isolates (62.2%) were identified as ESBL-producers. Multilocus sequence typing of the ESBL-producers identified 20 different sequence types (STs) with ST131 (n = 25, 24.5%) as the dominant group. Other detected STs included ST410 (n = 21, 20.6%) and ST617 (n = 19, 18.6%). All identified ESBL-producers harbored *bla*_CTX-M-14_, *bla*_CTX-M-15_, or *bla*_CTX-M-27_, with *bla*_CTX-M-15_ (n = 96, 94.1%) being the most predominant ESBL allele. Further analysis showed that the immediate genetic environment around *bla*_CTX-M-15_ is conserved within *bla*_CTX-M-15_ containing strains. Five of the 25 ST131 isolates were clustered with clade A, one with sub-clade C1, and 19 with the dominant sub-clade C2. The results show that fluoroquinolone-resistant, *bla*_CTX-M-14_- and *bla*_CTX- M-15_-producing ESBL *E*. *coli* ST131 strains belonging to clade A and sub-clades C1 and C2 are disseminating in Ghanaian hospitals. To the best of our knowledge, this is the first report of the ST131 phylogeny in Ghana.

## Introduction

Beta-lactam antimicrobials have been used extensively in healthcare settings and animal husbandry to treat bacterial infections. The spread of extended-spectrum β-lactamase (ESBL)-producing *Escherichia coli*, such as the high-risk clone ST131, has resulted in a global surge in the number of ESBL-producing *E*. *coli* strains after their first identification in 1983 in Germany^1,2^.

*Escherichia coli* ST131 is a pandemic strain responsible for numerous cases of recurrent urinary tract infections and sepsis^3^. Following its first identification in 2008, ST131 has attained global dominance, possibly primed by the sequential acquisition of certain virulence factors and antibiotic-resistant determinants^4–6^.

Proliferation of this pathogenic clone was demonstrated to be driven by a single subclone, H30^7,8^, also named clade C, which carries genes encoding the type 1 fimbriae adhesin. Given its multidrug resistance and association with ESBLs, ST131 is an urgent public health concern. Recently, resistance to carbapenems and colistin in ST131 clones was reported^9–11^. This phenomenon reduces therapeutic options, as these are last-resort antibiotics^11^.

Cefotaximase-15 beta-lactamase (*bla*_CTX-M-15_) is widely disseminated and is the most frequently identified ESBL allele in ST131^12^. Phylogenetically, ST131 is classified into three clades, A, B, and C. Clade A is the most divergent, antibiotic-sensitive clade associated with *fim*H41 and differs from B and C by approximately 7000 and 8900 single-nucleotide polymorphisms (SNPs), respectively^3,13^. Clade B, known to be involved in human-to-animal transmission, is typically fluoroquinolone-susceptible and differs from clade C by 70 nucleotide substitutions; and is identified primarily by *fim*H22^3,14^. The evolution of clade C was reported to coincide with the introduction and clinical use of fluoroquinolones (FQs) in the mid-1980s^15^. Strains in clade C possess FQ alleles that are consistent with their FQ-resistant phenotype^3^. Clade C also appears to be the most dominant group expressing the *fim*H30 allele^3^. This clade was further divided into nested sub-lineages C1/H30R and C2/H30Rx. C1/H30R is often associated with *bla*_CTX-M-14_/*bla*_CTX-M-27_, and C2/H30Rx is associated with *bla*_CTX-M-15_-type ESBLs, with the ESBL alleles typically carried on IncF plasmids^16–18^.

Previous studies demonstrated that other hypervirulent STs have not attained the same global prominence as ST131; however, the unique traits responsible for the global dissemination of ST131 remain unclear^13,19^. Although several studies have reported on the phylogeny of ST131 globally, data on ST131 in Ghana is scanty. Here we characterized 25 ST131 isolates from Ghana by whole genome sequencing. We investigated their genetic features and phylogenetic relationship with other globally described ST131 strains.

Findings from this study should provide insights into the role of insertion sequences and plasmids in promoting antimicrobial resistance and enable determinations of the extent of spread of various clades in Ghana.

## Results

### Antibiotic susceptibility profiles and characterization of ESBLs

Overall, *E. coli* isolates showed varying proportions of resistance to the antibiotics against which they were tested. The resistance ranged from 1.83% (amikacin) to 87.8% (sulfamethoxazole trimethoprim). Among the isolates, 104 (63.4%), 102 (62.2%), and 108(65.9%) were resistant to levofloxacin, cefotaxime, and ciprofloxacin, respectively. The rates of antibiotic resistance, MIC_50_, MIC_90_, and ranges of all isolates are shown in Table 1. All 102 (62.2%) CTX-resistant *E*. *coli* isolates were ESBL-producers; among them, 96 (94.1%) harbored *bla*_CTX-M-15_, five had *bla*_CTX-M-14_, and one had *bla*_CTX-M-27_. Among the 102 ESBL-producers, 90 (88.2%) had co-resistance to either ciprofloxacin or levofloxacin. Double mutations, serine 83 to leucine (S83L) and aspartic acid 87 to asparagine (D87N), were observed in the GyrA regions of 19 isolates, and the single mutation S83L was detected in the GyrA region of five *fim*H41 isolates, four of which carried *bla*_CTX-M-14_-ESBL (Supplementary data S1). All *bla*_CTX-M-15_ isolates with double mutations were associated with elevated MICs for fluoroquinolones, and especially ciprofloxacin (> 8 µg/mL), compared to the relatively low MICs (1 µg/mL) of fluoroquinolones for the *fim*-H41, *bla*_CTX-M-14_ ESBL strains (Supplementary data S2) with single mutations.

**Table 1:** Minimum inhibitory concentration 50 (MIC_50_) and Minimum inhibitory concentration 90 (MIC_90_) values and antibiotics resistance percentage values of 164 *E. coli* isolates.

Antimicrobial agents	Breakpoint for resistance (μg/mL)	% resistance	MIC (μg/mL)		
			Range	MIC_50_	MIC_90_
Piperacillin	≥ 128	86.0	1–64	> 64	> 64
Cefazolin	≥ 8	72.0	1–>16	> 16	> 16
Cefotaxime	≥ 4	62.2	≤ 0.5–> 32	32	> 32
Ceftazidime	≥ 16	48.8	≤ 0.5–> 16	8	> 16
Cefepime	≥ 16	50.6	≤ 0.5–> 16	16	> 16
Sulbactam/ampicillin	≥ 16/32	40.9	≤ 2/4–> 8/16	8/16	> 8/16
Cefpodoxime	≥ 8	62.2	≤ 1–> 4	> 4	> 4
Aztreonam	≥ 16	57.3	≤ 0.5–> 16	16	> 16
Imipenem	≥ 4	0.0	≤ 0.25–2	≤ 0.25	≤ 0.25
Meropenem	≥ 4	0.0	≤ 0.25–1	≤ 0.25	≤ 0.25
Gentamicin	≥ 16	39.0	≤ 0.25–> 8	1	> 8
Amikacin	≥ 64	1.83	≤ 1–> 32	4	16
Minocycline	≥ 16	42.1	0.5–> 8	8	> 8
Fosfomycin	≥ 256	0.0	≤ 32–64	≤ 32	≤ 32
Sulfamethoxazole/trimethoprim	≥ 76/4	87.8	≤ 9.5/0.5–> 38/2	> 38/2	> 38/2
Levofloxacin	≥ 2	63.4	≤ 0.25–> 4	> 4	> 4
Cefmetazole	≥ 64	2.4	≤ 0.5–128	2	8
Ciprofloxacin	≥ 1	65.9	≤ 0.25– > 8	> 8	> 8

### STs and fimH alleles

**Figure 1:**
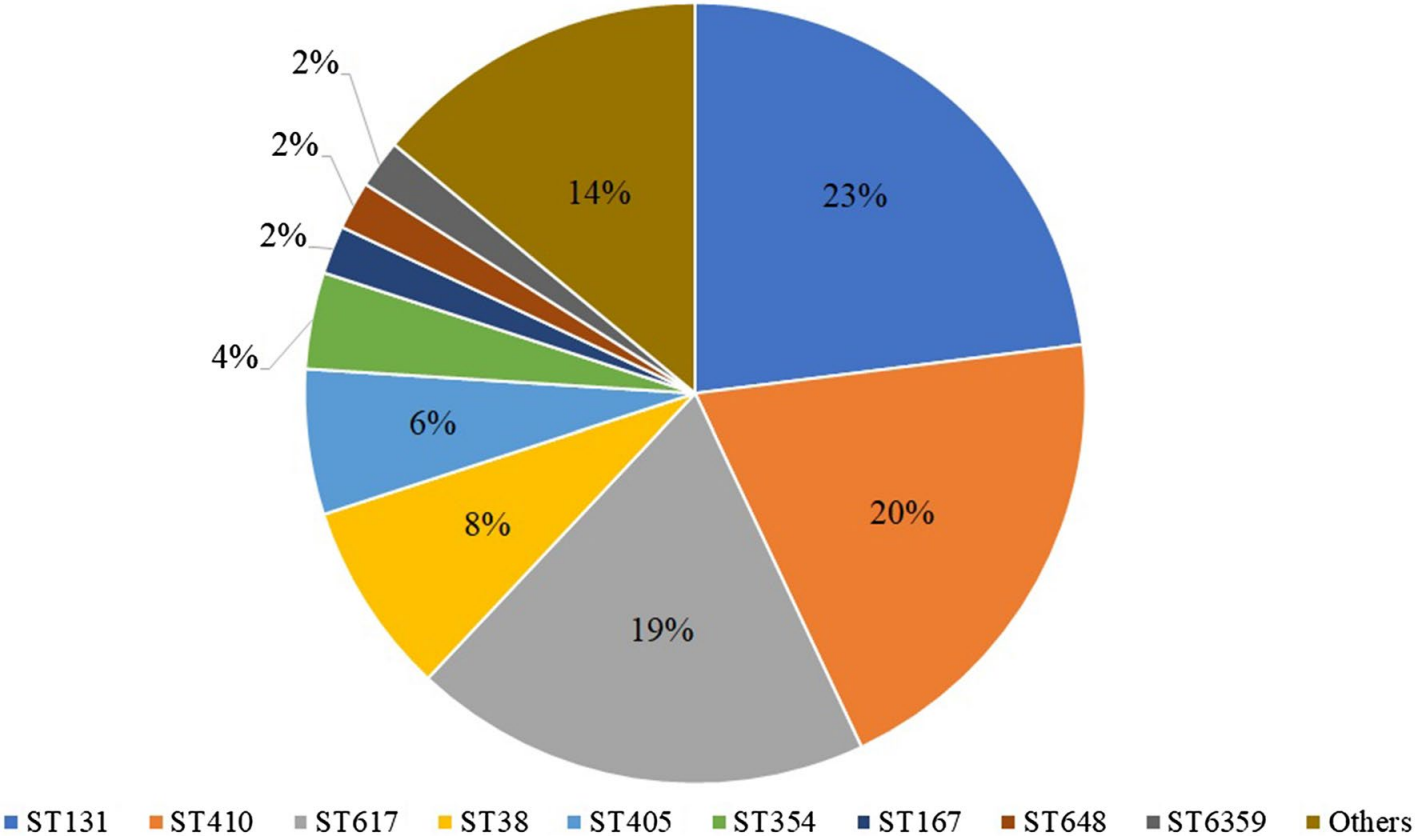
Distribution of sequence types (STs) among 102 extended-spectrum beta-lactamase (ESBL) producing *Escherichia coli* isolates. Isolates grouped as others belong to ST10, ST44, ST156, ST361, ST448, ST744, ST940, ST1303, ST1722, ST1727, and ST1788.

**Figure 2:**
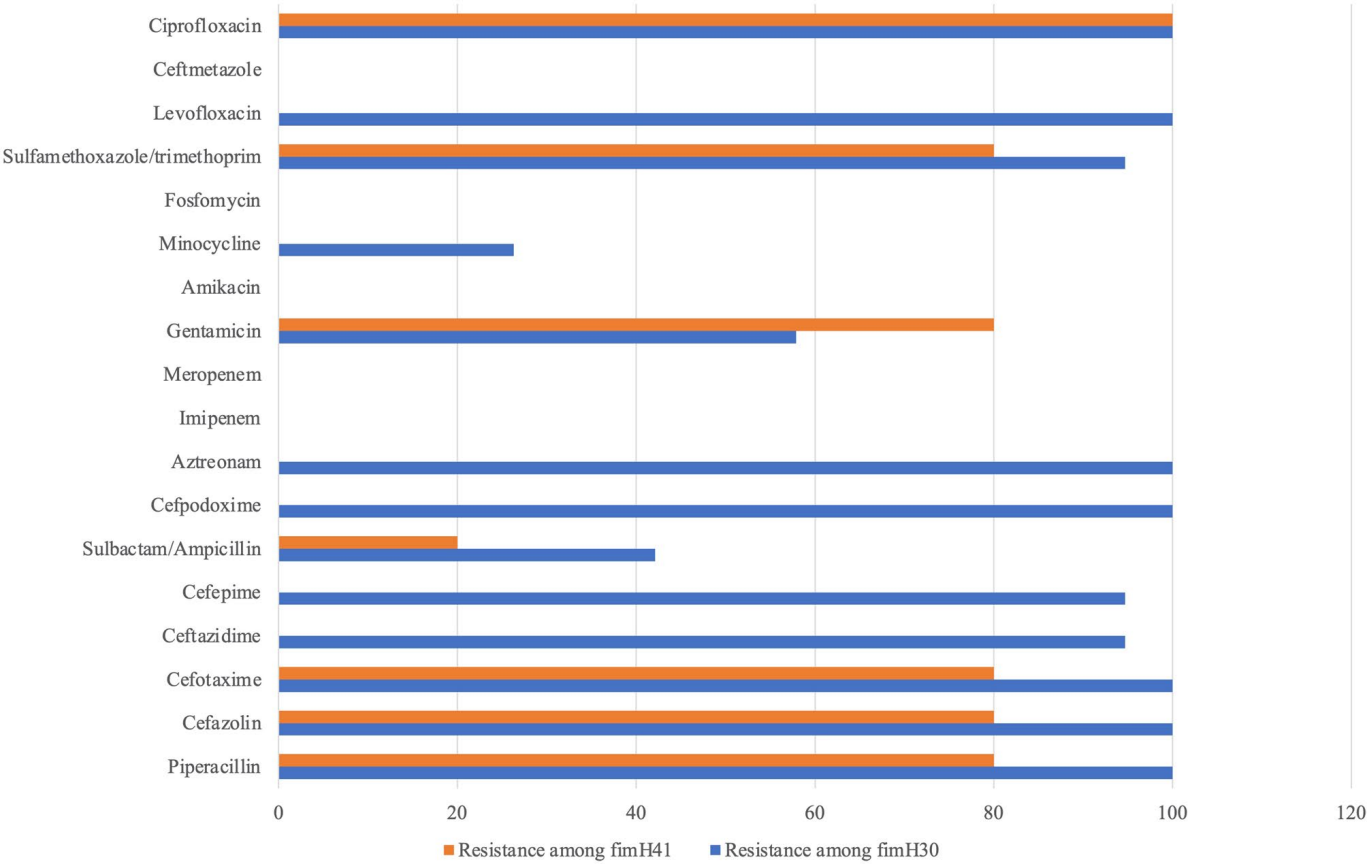
Antibiotic resistance amongst *fim*H30 and *fim*H41 ST131 strains. Percentages of *fim*H30 strains are shown in blue color and the percentages of *fim*H41 strains are shown in orange color.

The 102 ESBL-producers were classified into 20 sequence types (STs; Fig. 1). *E. coli* ST131 was predominant (n = 24, 23.5%), followed by ST410 (n = 21, 20.6%) and ST617 (n = 19, 18.6%). These three STs accounted for approximately three-fifths of the total number of ESBL-producers. Strains identified as ST131 showed high (100%) resistance rate to ciprofloxacin and were co-resistant to other antibiotics (Table 2). Among ST131 strains, we also observed that the *fim*H30-associated strains exhibited high resistance to antibiotics compared to the *fim*H41-bearing strains. However, considering reports about antibiotic sensitivity of *fim*H41 strains^3,13^, it is important to note that *fim*H41 strains showed 80% resistance to five antibiotics (Fig. 2). From the initial ST131 PCR screening, one non-ESBL and 24 ESBL isolates belonged to ST131. This non-ESBL producing isolate, also showed low-level resistance (MIC 1 µg/mL) to ciprofloxacin. Considering that ST131 strains are associated with fluoroquinolone resistance^20^, we included this non-ESBL strain in subsequent analysis after MLST confirmed that it was ST131, to compare ESBLs and non-ESBLs among ST131 strains. The *fim*H30 allele (n = 19, 76%) was the most common *fim*H type detected in 19 of 25 *E*. *coli* ST131 isolates. This was followed by *fim*H41 (n = 5, 20%) and *fim*H99 (n = 1, 4%). Four of the five isolates with *fim*H41 among ST131 harbored *bla*_CTX-M-14_.

**Table 2:** Minimum Inhibitory Concentration 50 (MIC_50_) and Minimum Inhibitory Concentration 90 (MIC_90_) values and antibiotics resistance percentage values among twenty-five (25) ST131 E. coli isolates.

Antimicrobial agents	Breakpoint for resistance(μg/mL)	% resistance	MIC (μg/mL)		
			Range	MIC_50_	MIC_90_
Piperacillin	≥ 128	96.0	1–> 64	> 64	> 64
Cefazolin	≥ 8	96.0	1–> 16	> 16	> 16
Cefotaxime	≥ 4	96.0	≤ 0.5–> 16	32	> 32
Ceftazidime	≥ 16	76.0	≤ 0.5–> 16	16	> 16
Cefepime	≥ 16	76.0	≤ 0.5–> 16	> 16	> 16
Sulbactam/ampicillin	≥ 16/32	36.0	≤ 2/4–> 8/16	8/16	> 8/16
Cefpodoxime	≥ 8	96.0	≤ 1–> 4	> 4	> 4
Aztreonam	≥ 16	80.0	≤ 0.5–> 16	> 16	> 16
Imipenem	≥ 4	0.0	≤ 0.25–≤ 0.25	≤ 0.25	≤ 0.25
Meropenem	≥ 4	0.0	≤ 0.25–≤ 0.25	≤ 0.25	≤ 0.25
Gentamicin	≥ 16	64.0	0.5–> 8	> 8	> 8
Amikacin	≥ 64	0.0	≤ 1–16	8	16
Minocycline	≥ 16	20.0	1–> 8	4	> 8
Fosfomycin	≥ 256	0.0	≤ 32–≤ 32	≤ 32	≤ 32
Sulfamethoxazole/trimethoprim	≥ 76/4	92.0	≤ 9.5/0.5–> 38/2	> 38/2	> 38/2
Levofloxacin	≥ 2	80.0	1–> 4	> 4	> 4
Cefmetazole	≥ 64	0.0	1–8	2	4
Ciprofloxacin	≥ 1	100.0	1–> 8	> 8	> 8

### Pangenome and phylogenetic analysis

**Figure 3:**
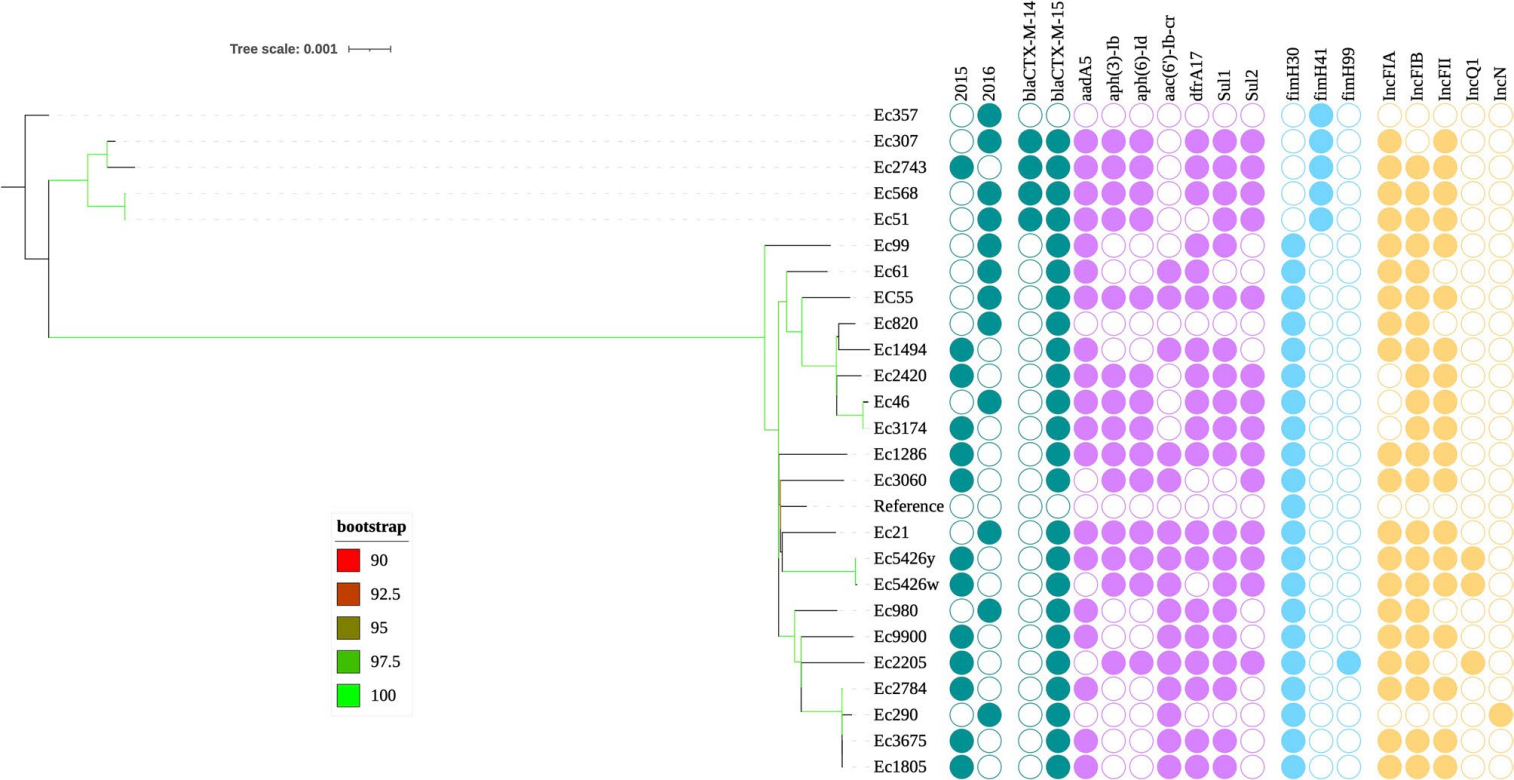
Rooted maximum-likelihood phylogenetic tree of 25 *Escherichia coli* ST131 isolates constructed based on 1037 core-genome alignment. Branch patterns were assessed by 1000 bootstrap replicates. Bootstrap support values are shown from 90 (minimum) to 100 (maximum). Year of isolation, presence of resistance genes, fim types, and plasmid replicon types are shown in a binary fashion as shaded circles with labels at the tips. Figure was visualized and annotated with ITOL v5 (https://itol.embl.de/personal_page.cgi).

An analysis of 25 ST131 isolates identified 7700 genes with 3926 core genes, 110 soft core genes in 95–99% of isolates, 1639 shell genes in 15–95%, and 2025 cloud genes in 15% of isolates. The genome sequence of the 25 strains were mapped onto that of reference strain EC958. A maximum likelihood tree was built on 1037 core genome SNP alignment. The strains were clustered into two major groups, the *fim*H41-associated group composed of five strains, and a *fim*H30 group with 20 strains (Fig. 3). In silico antibiotic resistance gene profiling showed that most isolates contained multiple resistance genes (Supplementary data S1, Fig. 3).

The plasmid replicon type IncFIB was the most common IncF-type plasmid, found in 21 (84.0%) isolates, followed by IncFIA 19 (76.0%) and IncFII 8 (32%). IncQ1 was detected in three (12.0%) isolates. IncN was assigned to one (4.0%) isolate (Supplementary data S1).

An analysis of 25 ST131 and 86 global ST131 isolates identified 11,942 genes. There were 3705 core genes in 99–100% of the isolates, 193 soft core genes in 95–99%, 1583 shell genes in 15–95%, and 6461 cloud genes in 15% of the isolates. Among the global collection of 86 isolates, 24 were isolated in the United States (US), 13 from the United Kingdom (UK), 13 from Germany, six from the Democratic Republic of Congo (DR Congo), four from Denmark, four from Spain, three from Japan, three from Australia, two from Tanzania, two from Ireland, two from Thailand, two from Canada, one from Portugal, one from Laos, one from Vietnam, one from the Netherlands, and one from Nepal. There was no information on the geographical locations of three isolates.

**Figure 4:**
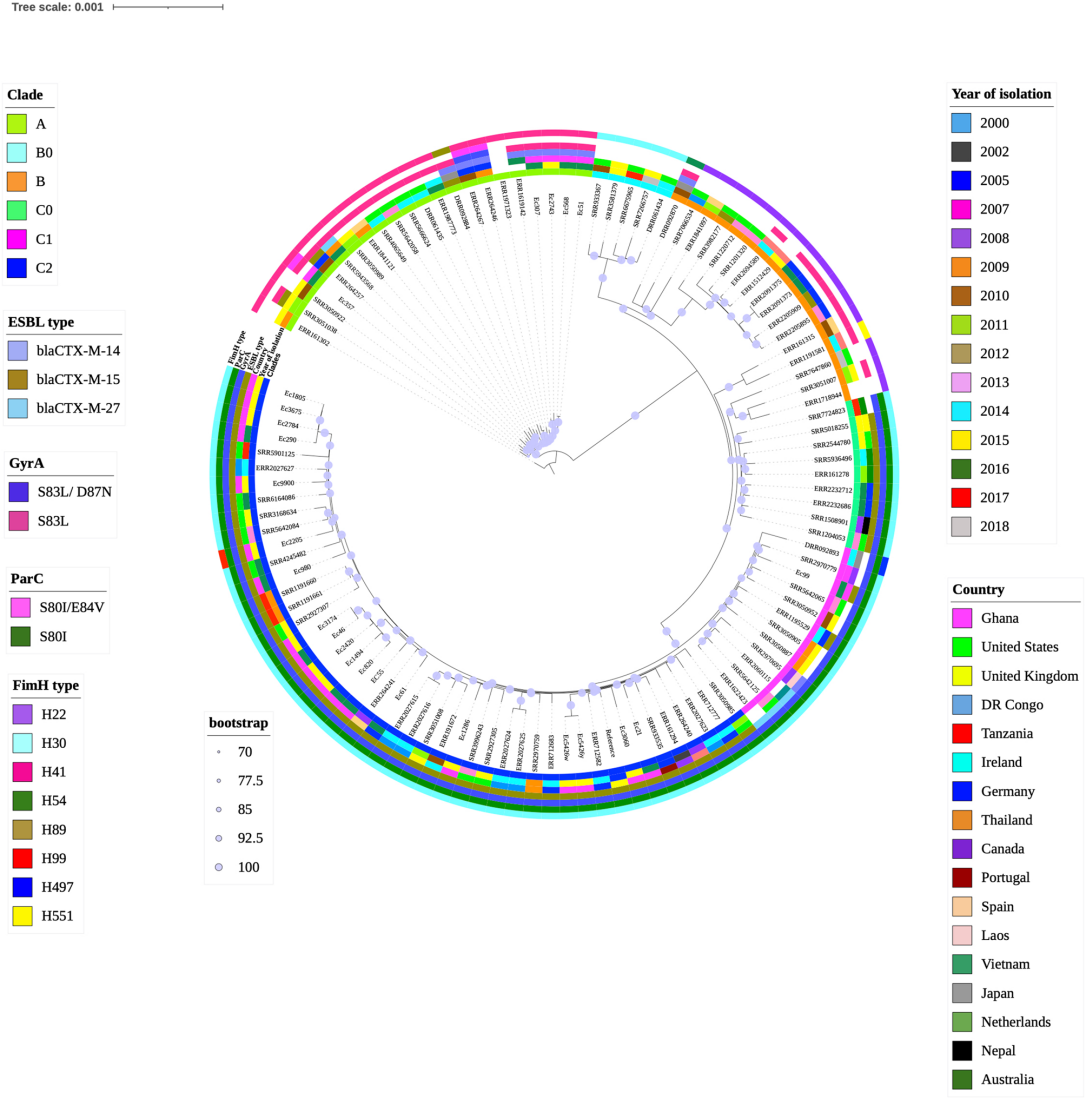
Rooted maximum-likelihood phylogenetic tree of 25 *Escherichia coli* ST131 and 86 *E. coli* ST131 representing the global collection. The phylogenetic tree was built based on 2656 core genome SNPs alignment. Branch patterns were assessed by 1000 bootstrap replicates. Bootstrap support values are shown from 70 (minimum) to 100 (maximum). Clades A, B0, B, C0, C1, and C2 are represented by strips colored lemon green, turquoise, orange, light green, pink, and blue, respectively. The year and country of isolation are displayed in assorted colored strips. Allelic profiling data are shown as color strips from inside out as the ESBL type, GyrA, ParC, and *fim* type. Accession numbers and IDs of the strains are labeled at the tips. Figure was visualized and annotated with ITOL v5 (https://itol.embl.de/personal_page.cgi).

All 86 isolates, together with the 25 isolates from Ghana, classified into six ST131 subclades, A, B0, B, C0, C1, and C2. The dominant subclade observed was C2. Nineteen of the Ghana isolates clustered with strains from various geographic regions in clade C2. One isolate clustered with other strains in clade C1 and five clustered with strains in clade A. None of our isolates clustered with others in C0, B, and B0. Some clustered closely with strains from the US, Ireland, Spain, the UK, Germany, and Australia. Notably, although the strains from other African countries (Democratic Republic of Congo, Tanzania) clustered among the C2 group, none clustered closely with our Ghanaian isolates (Fig. 4).

### Genetic environment of ***bla***_CTX-M-15_

**Figure 5:**
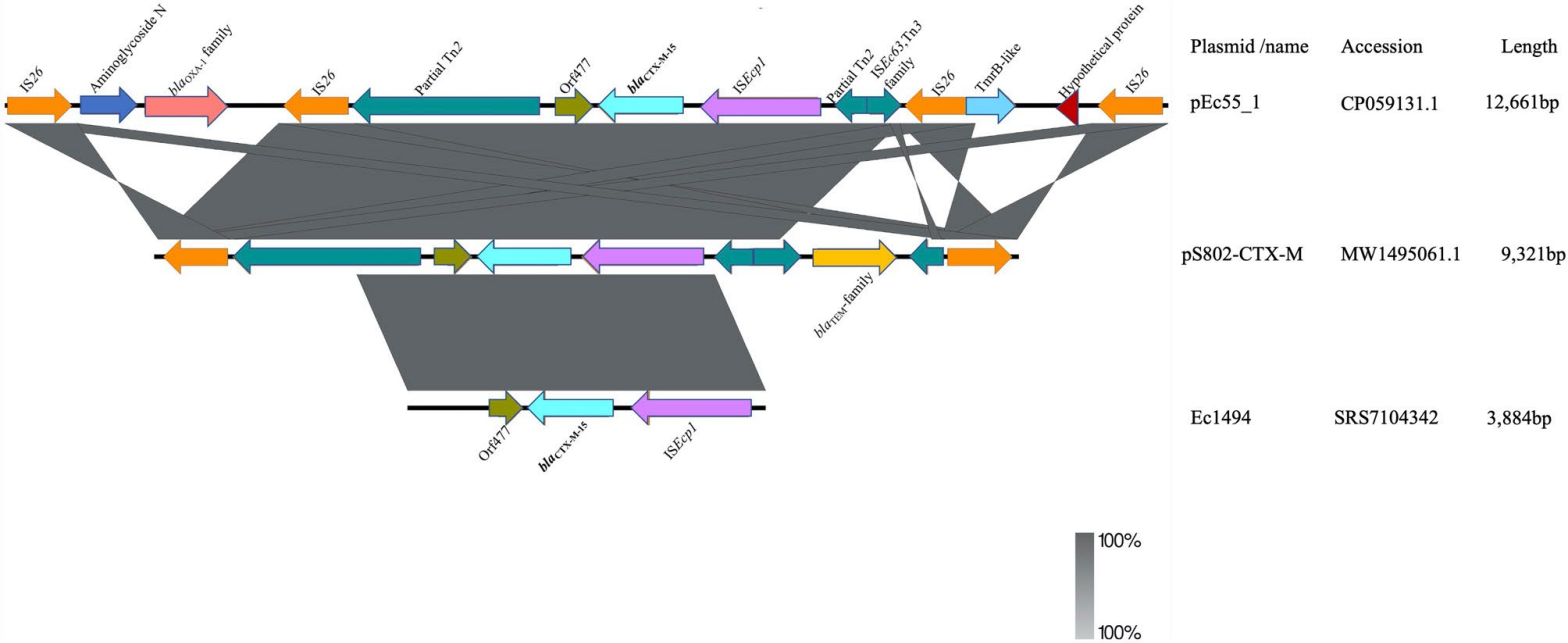
Comparison of the genetic environment of *bla*_CTX-M-15_. Arrows depict the genes and sequences upstream and downstream of *bla*_CTX-M-15_ in their transcriptional directions as detected by sequencing. The structure of pS802-CTX-M was reversed to enable easy comparison. Shaded regions indicate regions that are common to all isolates. Figure was created and visualized with Easyfig v2.2.2 (https://mjsull.github.io/Easyfig/files.html).

*bla*_CTX-M-15_ was found on a 167,739 bp length IncFII plasmid pEc55_1 (accession no. CP059131.1) in strain EC55, flanked by intact IS*Ecp1* with 48 bp spacer upstream and an open reading frame 477 (ORF477) which encodes tryptophan synthase, with a 46 bp spacer downstream. This structural arrangement was observed in strains Ec1494 and the 162,792 bp plasmid pS802-CTX-M. However, *bla*_CTX-M-15_ was found on a 3,015,999 bp length in Ec1494, suggesting *bla*_CTX-M-15_ may have been integrated into the chromosome of Ec1494. In pEC55_1 and pS802-CTX-M, these were also flanked by two partial Tn*2* transposons truncated by two complete, IS*26* insertion sequences further upstream and downstream of *bla*_CTX-M-15_. The partial Tn*2* is truncated by a gene belonging to the *bla*_TEM_ family in pS802-CTX-M, while a *bla*_OXA_ gene, which might have been mobilized by an adjacent IS*26*, was found lying downstream of *bla*_CTX-M-15_ between two IS*26* insertion sequences in pEC55_1. Compared to pEC55_1 and pS802-CTX-M, a partial Tn*2* was found only downstream of *bla*_CTX-M-15_ in Ec1494 (Fig. 5).

## Discussion

Our study provides information on the epidemiology, antibiotic resistance, and phylogenetic relationships among ST131 isolates from Ghana. We documented the highest rate of resistance to sulfamethoxazole trimethoprim. This rate was relatively higher than that reported (78.4%) by Freeman et al., also in Ghana^21^, and comparably lower than that reported in a Nigerian study, in which more than 90% of the isolates were resistant to trimethoprim sulfamethoxazole^22^. Recent studies in Ghana show that antibiotics are heavily abused in Ghanaian communities and among tertiary students. The research disclosed that individuals who engaged in this practice resort to self-medication partly, as a result of financial constraints, as most could barely afford the cost of proper medical care, even when they have knowledge of the implications; moreover, some of these individuals fail to complete the full course of their antibiotic treatment^23,24^. Additionally, data from previous studies show that antibiotics are prescribed and dispensed particularly in the rural areas of Ghana by unauthorized personnel^23,24^. The consequences of such practices are the observed clinical resistance to antibiotics and treatment failure due to selective pressure. In light of this, more stringent measures need to be instituted to regulate prescriptions and the usage of antibiotics in Ghana.

More than half of the isolates were frequently resistant to third-generation cephalosporin, cefpodoxime (62.2%), whereas 48.8% were resistant to ceftazidime. A study of the antibiotic prescription rate in Ghana revealed that cephalosporins are highly prescribed because of the availability of inexpensive generic forms that are covered by the National Health Insurance Scheme (NHIS) in Ghana^25^. Cephalosporins are also preferred for empirical therapy because of their minimal toxicity^25^. The extensive use and high resistance rate of these antimicrobial agents can result in a serious therapeutic challenge in the future if left unchecked.

In most laboratories, cefotaxime is broadly used to identify ESBL-producing isolates ^26^. Here, 102 (62.2%) isolates were resistant to cefotaxime. These harbored *bla*_CTX-M-14_, *bla*_CTX-M-15_, or *bla*_CTX-M-27_. Consequently, the observed resistance is likely attributed to the presence of *bla*_CTX-M_ genes. All isolates in our collection were susceptible to fosfomycin, meropenem, and imipenem. These medicines are costly, not covered by the NHIS in Ghana, and are used to treat serious infections; therefore, their prescription rate is extremely low^25^ and this might explain the 100% susceptibility observed in this study.

More than half (62.2%) of the isolates were ESBL-producers, contradictory to the relatively low prevalence (16.1%) among 143 extraintestinal pathogenic (ExPEC) strains in a Tanzanian study^27^, where, the majority (128) of the patients were pregnant women and 15 children. The results also showed that ESBL production was significantly increased in children than in the pregnant women, possibly resulting from hospital-acquired bloodstream infections among the children. Much of the difference between the ESBL prevalence in both studies could be due to the patient group involved, which also points to the possible dissemination of ESBL strains in hospital settings and the need to implement robust systems to control the situation. Another study^28^ in Ghana detected 62% of ESBL-producing isolates, similar to the result from this study. This high occurrence of ESBLs among clinical isolates is of concern as it is likely to result in reliance on last line antibiotics for treatment and the development of new antibiotics may not keep pace with development of resistance in bacteria, plunging us into a post-antibiotic era^29^.

Most ESBL-producing *E*. *coli* have FQ resistance, through chromosomal mutations of FQ-target sites^30–32^. Like previously reported results for FQ resistance, we found that multiple substitutions in target sites cause high-level ciprofloxacin resistance among *bla*_CTX-M_-producing *E*. *coli*. *E. coli* ST131 is a global pandemic clone, and clade C2/fimH30-Rx, in particular, is known to be highly virulent^15,33,34^. In this study, although the *bla*_CTX-M_-producers were diverse, *E*. *coli* ST131 was the prime genotype among FQ-resistant, *bla*_CTX-M_-producing *E*. *coli*, similar to previous reports from Ghana^35^ and Nigeria^36^. Specifically, the fimH30-Rx clone appears to have spread widely in Ghanaian hospitals, as it has in many different regions worldwide^34^. The prevalence of ST131 (23.5%) among ESBL-producing *E. coli* was comparable to the 20.5% in a study in Europe^37^; but lower than the prevalence from human isolates (52%) in a Canadian study^38^. The differences in ST131 prevalence among countries has been attributed to factors such as the antibiotic consumption rate and dietary habits^37^. This study also reports a high number of FQ-R *bla*_CTX-M-15_ producing ST410 and ST617 strains. ST410 has been recently described as a high-risk clone with multi-drug resistance like ST131, coupled with a wide host range^39^. Also, ST410 comprises ESBL and carbapenem-resistant strains, as has been reported of the globally disseminated NDM-positive *E. coli* strains of ST617^40^. The detection of such high-risk clones heightens the need for regular monitoring of drug-resistant clones.

Among the CTX-M-type genes of ESBL-producing *E*. *coli*, *bla*_CTX-M-15_ and *bla*_CTX-M-14_ are the predominant subtypes worldwide; however, data on the prevalence of these genes in Ghana are limited. The results show that *bla*_CTX-M-15_ is the most widely disseminated CTX-M-type genes in Ghana among ESBL-producing *E*. *coli*, which agrees with previously published data^28,41,42^. SNP-based phylogenetic analysis grouped the 25 isolates into two major groups corresponding with their *fim*H alleles. Strains with *fim*H41, otherwise known as clade A isolates, formed a distinct cluster from the *fim*H30 isolates. A limitation of this study is that isolates were collected from only two hospitals, one of which is a teaching hospital. We could not get information on the clinical diagnosis and specimens from which some of the isolates were recovered. Analysis of such data would have added to current knowledge on the dissemination of resistant strains.

Phylogenetic analysis clustered the Ghanaian strains closely with some international strains, and most (19/25) belonged to clade C2, a few (5/25) belonged to clade A, and one belonged to clade C1. This shows the extent to which clade C, particularly C2, is disseminated worldwide. All clade C isolates were also characterized as *fim*H30. All five clade A isolates had *fim*H41.The association among these fim types suggests that the clades are defined by their fim types.

In bacteria, the plasmid-mediated transfer of drug-resistance determinants widely contributes to the dissemination of antimicrobial resistance^26^. IncFII particularly is suggested to have played a central role in the emergence of C2/H20Rx clade of the ST131^19^. The results showed that *E. coli* ST131 isolates harbored plasmids predominantly with replicon type F. This is noteworthy and reinforces the pivotal role of IncF plasmids in the dissemination of antibiotic resistance in Enterobacteriaceae. The results also corroborate the findings of other studies in which IncF plasmids were frequently recovered from *E*. *coli* isolates^43–45^. IncF replicon detection also agreed with the results of other reports from Tanzania based on a study where plasmids were detected in *E*. *coli* isolates from drinking water sources^46^ and a study from Nigeria detecting *E*. *coli* isolates from humans and bovine sources^47^. All isolates studied harbored IncF plasmids carrying the ESBL genes *bla*_CTX-M-15_ or *bla*_CTX-M-14_. There is a postulation that a fluoroquinolone resistant C1 strain obtained ancestral IncFII plasmid and eventually integrated a *bla*_CTX-M-15_ transposition unit, which has been incorporated into the chromosome through transpositional events and recombination within the C2/H30Rx clade^19^.

In agreement with other studies^45,46^, IncFIB, 21 (84.0%) was the most identified replicon type and was almost always associated with *bla*_CTX-M-15_, followed by IncFIA (76.0%) and IncFII (32.0%). These results follow the same pattern reported by other authors^28^ for *E*. *coli* isolates. Previous studies reported *bla*_CTX-M-15_ as part of a 2971 bp transposition unit typically found 48 bp to the right of the inverted repeat side of IS*Ecp1* with a partial ORF (ORF477), and this transposition unit is inserted in Tn*2*^48^. The extent to which *bla*_CTX-M_ genes are expressed have also been shown to be highly dependent on the length of the spacer sequence between IS*Ecp1* and the start codon of *bla*_CTX-M_ genes^49^. Notably, in the isolates investigated, IS*Ecp1* was found 48 bp upstream of *bla*_CTX-M-15_. This arrangement has also been reported in studies in France, Canada, Italy, the UK, and Spain^50–52^. The evolutionary relationship between *bla*_CTX-M-15_ and IS*Ecp1* has also been reported in several countries worldwide^53^. IS*Ecp1* belongs to the insertion sequence family IS*1380* and has been established as one of several factors involved in mobilizing *bla*_CTX-M_ genes^53^. Transposon sequences in the pEc55_1 was disrupted by IS*26* upstream and downstream of *bla*_CTX-M_ genes. This association shows the magnitude of the impact of IS*26* on shaping antibiotic resistance regions in gram-negative bacteria^53^. According to the results of previous studies, the minimum inhibitory concentrations of cefepime and ceftazidime in isolates with *bla*_CTX-M-15_ preceded by IS*26* is higher than those in isolates without IS*26*.

## Conclusion

A comparative analysis was used to describe the genetic features of 164 *E. coli* isolates from two hospitals in Ghana. A high percentage (62%) of the isolates was found to produce ESBL, and these were classified into 20 MLST STs. The pandemic clone ST131 was the dominant ST found. This study also reports a conserved genetic environment for the most prevalent *bla*_CTX-M-15_ gene, whether plasmid associated or chromosomal. Phylogenetic analysis revealed that our isolates and other African isolates clustered together with other international strains among clades C, and particularly C2 and A, indicating that same ST131 clone is disseminating worldwide. The detection of FQ-resistant ESBL-producing ST131 shows that ST131 might be circulating in hospitals in Ghana and calls for constant monitoring to control the spread of this highly pathogenic clone. To the best of our knowledge, this is the first report of ST131 that details a SNP-based core genome phylogeny and appraises the epidemiology of its clades and subclades in Ghana.

## Materials and methods

### Ethical consideration

The Institutional Review Board of the Noguchi Memorial Institute for Medical Research, University of Ghana (FWA00001824) and the Faculty of Medicine, Tokyo Medical and Dental University (M2017-208) reviewed and approved all protocols for this study. Written informed consent was obtained from all participants of the study. All experiments were performed in accordance with the relevant guidelines and regulations.

### Bacterial isolation and identification

In total, 164 non-duplicate *E*. *coli* isolates recovered from clinical specimens of urine (n = 77), high-vaginal swab HVS (n = 20), wound swabs (n = 11), sputum (n = 9), stool (n = 3), endocervical swabs (n = 3), semen(n = 3), ascitic fluid (n = 1), an ear swab (n = 1), pus (n = 1), urethral discharge (n = 1), and others (n = 34) were obtained from the Effia Nkwanta Regional Hospital and Tamale Teaching Hospital between March 2015 and April 2016. The isolates were stored in 10% skimmed milk at − 80 °C prior to testing and were identified using a MALDI Biotyper (Bruker Daltonics, Billerica, MA) and VITEK MS (bioMérieux, Marcy-l’Étoile, France).

### Antimicrobial susceptibility testing

The minimum inhibitory concentrations of 16 of 18 antibiotics, specifically piperacillin, cefazolin, cefotaxime, ceftazidime, cefepime, sulbactam/ampicillin, cefpodoxime, aztreonam, imipenem, meropenem, gentamicin, amikacin, minocycline, fosfomycin, sulfamethoxazole trimethoprim, and levofloxacin, were determined by broth microdilution on a DP31 dry plate (Eiken Chemical Co., Tokyo, Japan). The minimum inhibitory concentrations of the other two antibiotics, cefmetazole and ciprofloxacin, were determined by the agar dilution method, and the results were interpreted according to the Clinical Laboratory Standards Institute (CLSI) guidelines M100-S30. The experiment was controlled using the *E. coli* reference strain ATCC 25,922 for negative quality control. ESBL phenotypes were confirmed using *Klebsiella pneumoniae* ATCC 700603 as a positive quality control strain according to the CLSI guidelines M100-S30.

### Characterization of ESBL genes, ST131 clade screening, and multi-locus sequence typing

DNA was extracted using the Cica Geneus™ DNA Extraction reagent (Kanto Chemical Co., Tokyo, Japan). PCR was conducted to determine the genetic basis of resistance in ESBL-producers, targeting β-lactamase-encoding genes (*bla*_TEM-_, 800 bp; *bla*_SHV-_, 713 bp; *bla*_CTX-M,_ 688 bp; *bla*_OXA-1-like_, 564 bp), as previously described^54^. The presence of *bla*_CTX-M-15_ (band size 875 bp) was confirmed as previously described^55^. PCR was conducted to identify *bla*_CTX-M-14_ and *bla*_CTX-M-27_ using primer sets *bla*_CTX-M-group9-F_ (5′-ATGGTGACAAAGAGAGTGCAA-3′) and *bla*_CTX-Mgroup9-R_ (5′-CCCTTCGGCGATGATTCT-3′). Amplification was carried out with an initial denaturation at 94 °C for 4 min, followed by 30 cycles of 94 °C for 1 min, 55 °C for 1 min, and 72 °C for 1 min, and final extension at 72 °C for 10 min. PCR could not sufficiently discriminate between *bla*_CTX-M-14_ and *bla*_CTX-M-27_ because the band sizes for both *bla*_CTX-M-14_ and *bla*_CTX-M-27_ were 870 bp and therefore Sanger sequencing was employed, and the resulting amplified DNA sequences obtained were compared with those in the repository database using the BLAST program of the National Center for Biotechnology Information to distinguish between the genes. Isolates were initially screened for ST131 strains as previously described^56^.

Multi-locus sequence typing of all ESBL isolates was performed as described by Lau et al.^5^. Seven housekeeping genes (*adk*, *fumC*, *gyrB*, *icd*, *mdh*, *purA*, and *recA*) were amplified, sequenced, and an allele number was subsequently allocated according to EnteroBase (https://enterobase.warwick.ac.uk/). The STs were assigned based on the allelic profiles.

### Whole genome sequencing and assembly

ST131 isolates detected were subjected to whole-genome sequencing. Genomic DNA was extracted using the Nucleospin Tissue Kit (Macherey–Nagel, Düren, Germany). DNA (400 ng) was fragmented and subjected to size selection with an insert size of about 400 bp. For sequencing on DNBSEQ-G400FAST, DNA library was prepared using the MGIEasy FS PCR-Free DNA Library Prep set (MGI, Cat. No. 1000013454). DNA end repair and adapter ligation were conducted using the MGIEasy PF Adapters-96 Kit (MGI, Cat. No. 1000013454) following the manufacturer’s instructions. Finally, 75 fmol DNA nanoballs were prepared by combining 50 barcoded samples. The library was sequenced on the DNBSEQ-G400FAST (Tokyo, Japan) for short reads. Reads generated were filtered for quality checks such that low-quality reads (≤ Q30), short reads (≤ 10 bp), and accidentally residual adapter sequences were removed. The genomes of two strains (EC55 and Ec1494) were further sequenced on the Nanopore MinION (Oxford Nanopore Technologies, Oxford, UK) for long-read sequences to obtain complete genomes. Libraries were prepared using the SQK-LSK109 ligation sequencing kit (Oxford Nanopore Technologies, Oxford, UK). Low-quality reads (≤ Q10) and long reads (≤ 1000 bp) were filtered out. A de novo assembly was run using the assembler unicycler with the de Brujin graph, and the number of contigs obtained for all samples was 100–200. Hybrid assembly generated five contigs for strain EC55 (accession nos. CP059130.1–CP059134.1), with a chromosome length of 5,216,731 bp and four circular plasmids ranging between 1 and 16 kb. Thirty-seven contigs were generated from the hybrid assembly of sequences of strain Ec1494. The sequences outputted by unicycler was used for all analyses. Sequences were annotated using the online systems RAST and BLAST searches.

### Bioinformatics and phylogenetic analysis

Assembled sequences of the 25 Ghanaian strains were aligned to the complete genome sequence of reference strain EC958 (accession no: HG941718). SNPs were predicted, and the alignment file was cleaned using snippyv4.6.0^57^. Recombinant regions were excluded using Gubbinsv2.4.1^58^. Core genome SNPs were extracted using SNP-sites^59^ and a phylogenetic tree of 25 ST131 isolates was constructed using iq-tree^60^. Pangenome analysis was performed using Roary v3.12.0^61^. To investigate these isolates within the global context, we constructed a maximum-likelihood phylogenetic tree of all 25 isolates along with a global collection of 86 isolates from a published article^62^ based on 2656 core genome SNP alignment. Data on these 86 strains were obtained in December 2020. The raw reads were retrieved from GenBank and assembled with a unicycler. Assembled genomes were annotated using prokka v1.13^63^ software. All the genomes were mapped onto EC958, and variants were called. All genomes were processed using same procedure as for the 25 strains described above. The confidence of branching patterns was assessed using 1000 bootstrap replicates. The trees were visualized using iTOL v5^64^. Resfinder v3.3 was interrogated for acquired resistance genes and chromosomal point mutations. Insertion sequences were determined using ISFinder (https://isfinder.biotoul.fr/). Fim types were assigned using FimTyper (https://cge.cbs.dtu.dk/services/FimTyper/). Plasmid replicon types were determined using PlasmidFinder (https://cge.cbs.dtu.dk/services/PlasmidFinder/). To investigate the genetic context of *bla*_CTX-M-15_, we did a search of the *bla*_CTX-M-15_ region of our strain EC55 using BLAST. The search produced matches with several genomes in the database. We obtained and compared the genetic environment of *bla*_CTX-M-15_ in our strains EC55 and Ec1494 to one found on plasmid pS802-CTX-M (accession no: MW495061), recovered from the database with 100% query cover and sequence identity, and visualized using Easyfigv2.2.2^65^. Complete and draft genome sequences have been deposited in GenBank under the Bioproject PRJNA473419. Accession numbers of all strains used in this study can be found in Supplementary data S1.

## Supplementary Information

Supplementary Information 1.

Supplementary Information 2.

## Data Availability

Datasets generated during this study are available from the corresponding author upon reasonable request.
